# Ornithine α-Ketoglutarate Alleviates Inflammation *via* Regulating Ileal Mucosa Microbiota and Metabolites in Enterotoxigenic *Escherichia coli*-Infected Pigs

**DOI:** 10.3389/fnut.2022.862498

**Published:** 2022-06-07

**Authors:** Yuying Li, Xuetai Bao, Fan Yang, Junquan Tian, Wenxuan Su, Jie Yin, Kang Yao, Tiejun Li, Yulong Yin

**Affiliations:** ^1^Key Laboratory of Agro-Ecological Processes in Subtropical Region, Institute of Subtropical Agriculture, Chinese Academy of Sciences, National Engineering Laboratory for Pollution Control and Waste Utilization in Livestock and Poultry Production, Hunan Province Key Laboratory of Animal Nutritional Physiology and Metabolic Process, Changsha, China; ^2^College of Advanced Agricultural Sciences, University of Chinese Academy of Sciences, Beijing, China; ^3^College of Animal Science and Technology, Hunan Agricultural University, Changsha, China

**Keywords:** ornithine α-ketoglutarate, enterotoxigenic *Escherichia coli*, ileal mucosa microbiota, ileal mucosa metabolites, immune status, inflammation

## Abstract

Enterotoxigenic *Escherichia coli* (ETEC) is one of the main causes of diarrhea in weaned piglets, and ornithine α-ketoglutarate (OKG) as a food supplement has been shown to improve intestinal immune status in animals and humans. However, it remains unknown whether OKG alleviates inflammation through the regulation of gut microbiota and its metabolites on ETEC-infected piglets. This study was conducted to explore the impact of OKG on growth performance, immunity, and ileal mucosa microbiota and its metabolites in piglets infected with ETEC. On a total of 40 pigs, a 2 × 2 factor design was performed; the major factors were diet (basal diet or 1% OKG diet) and challenge (*E. coli* or LB Broth). The results showed that ETEC-infection inhibited growth performance, and OKG supplementation alleviated growth performance. Interestingly, ETEC-infection increased the serum TNF-α and IL-6, decreased the serum IL-10, downregulated the mRNA expression of IL-1β, IL-6, MyD88, and improved the mRNA expression of IL-8, IL-18, and TLR4. OKG inhibited serum IL-6, suppressed the phosphorylation of downstream signals of NF-κB/JNK in the ileum, and enhanced serum IL-10 and ileum SIgA in ETEC-challenged piglets. OKG supplementation enhanced the mRNA expression of IL-1β and IL-10 and reduced NF-κB and MyD88 in the ileum. Importantly, OKG reversed intestinal microbiota dysfunction, including the diversity of ileal microbiota, the relative abundances of *Actinobacillus*, *Turicibacter*, and *[Acetivibrio]_ethanolgignens_group*, which significantly affected arachidonic acid metabolism and primary bile acid biosynthesis. Collectively, our results suggest that OKG improves growth performance, regulates immunity, and ileal mucosa microbiota and its metabolites in ETEC-infected piglets.

## Introduction

Early weaned pigs generally exhibit hypoplasia of the immune system, disorder of the digestive system, and diarrhea after weaning (1). Neonatal and post-weaning piglets raise the opportunity for digestive pathogens, such as enterotoxigenic *Escherichia coli* (ETEC), to invade or colonize the gut. ETEC is a universal intestinal inhabitant for diarrhea in young animals, resulting in significant growth retardation and economic losses in pig production due to severe diarrhea, morbidity, mortality, and impaired growth during weaning transition (2, 3). ETEC K88 is often colonized in the small bowel and continuously secretes enterotoxins that damage the functions of the intestinal epithelium and triggers inflammation *via* increasing cell cation exchanges and reducing water absorption (4, 5). To solve the above problems, antibiotics are widely used to treat pathogen infections (6). In livestock, however, the overuse of antibiotics has ultimately led to serious problems, such as drug-resistant bacteria (7). Therefore, an effective nutritional feed ingredient is necessary to improve health status and inhibit diarrhea.

Ornithine α-ketoglutarate (OKG) is a nutritional compound that consists of one molecule of α-ketoglutarate and two molecules of ornithine, a precursor of glutamine, arginine, and proline, which benefit the immune response by altering inflammatory processes during times of challenge (8–12). More specifically, OKG improves glutamine in blood and has antitumor functions in combination with the glutaminase 1 enzyme pathway inhibitors (8). In recent years, OKG in stock farming has attracted growing attention. For instance, *in ovo*, 0.2 or 0.4% of OKG injection can promote early growth and pectoral muscles of chicks (8). Administration of 0.4 g/kg of OKG improves bone properties and amino acid synthesis in rapidly growing turkeys (13). Interestingly, our previous study found that 0.5 or 1% of OKG alters pig gut microbe, especially decreases the *Proteobacteria* (including *Escherichia_coli*), increases the serum of glutamate, proline, aspartate, threonine, and branch chain amino acid levels, and alleviates growth-suppression induced by D-galactose chronic oxidative stress (14). Dietary 0.75% OKG increases daily gain and feed intake in weaning pigs (15). Also, OKG (0.5, 1.5, and 4.5 g/kg/day) increases tissue glutamine concentration and N balance in endotoxemia rats (12). A total of 10 g/day OKG can cure pressure ulcers in elderly patients (11). The beneficial role of OKG in various physiological benefits has been validated, but the anti-inflammatory effect of OKG in intestinal inflammation was rarely revealed. Therefore, we aimed to explore the possible mechanism of the OKG in alleviating inflammation and regulating intestinal microbiota.

## Materials and Methods

### Animals and Diets

Forty healthy piglets (Duroc × Landrace × Yorkshire, half-castrated males and half females, average body weights of 8.25 kg) weaned at 28 days were used. After 3 days of adaption, all pigs were fed a standard diet. Then, the pigs were randomly assigned to either a basal (CON) or 1% OKG (*n* = 20/diet, NRC2012, Table 1). Each group of animals was randomly assigned to two sub-groups (ETEC and OKG ETEC; *n* = 10/treatment group). Pigs in the ETEC and OKG ETEC received orally 10 ml (2 × 10^9^ CFU/ml) of the enterotoxic strain of *E. coli* K88, respectively, whereas pigs in the CON and OKG groups were given an equal volume of LB Broth on day 15. The piglets were housed individually and had free access to diets and water. Three days after the challenge, all animals were euthanized. Samples of serum, ileum, ileal mucosa, and feces were immediately frozen at −80°C for analysis. All animal procedures were approved by the Animal Care Committee of the Institute of Subtropical Agriculture, Chinese Academy of Sciences.

**TABLE 1:** Ingredients and nutrient levels of the basal diet (%).

Item composition	Basal diet	OKG diet
Corn	62.51	62.51
Soybean meal	17.80	17.30
Fish meal	6.00	5.50
Wheat bran	0.30	0.30
Whey powder	2.63	2.63
Glucose	3.00	3.00
Soybean oil	3.00	3.00
Lysine	0.80	0.80
Methionine	0.46	0.46
Threonine	0.40	0.40
Tryptophan	0.10	0.10
CaHPO_4_	1.20	1.20
Limestone	0.50	0.50
NaCl	0.30	0.30
^1^Premix	1.00	1.00
OKG	0	1.00
Total	100.00	100.00
**Nutrient levels^2^**		
DE (MJ/kg)	14.69	14.53
CP	16.99	17.34
Lys	1.54	1.54
Met	0.86	0.86
Thr	0.95	0.95
Trp	0.25	0.25
Ca	0.78	0.78
TP	0.64	0.64
AP	0.42	0.42

*^1^Premix provided the following: vitamin 0.1, choline 0.16,CuSO_4_•H_2_O 0.05, MnSO_4_•H_2_O 0.03, ZnSO_4_•H_2_O 0.03, FeSO_4_•H_2_O 0.06, 1% iodine 0.001, 1% selenium 0.001, 1% cobalt 0.001, zeolite powder 0.567.*

*^2^Nutrient levels were calculated values.*

### Growth Performance and Feces Score

The body weight was recorded on days 0, 14, and 17, the feed intake was recorded daily to calculate average daily feed intake, and the feces score was recorded 0, 24, and 48 h after ETEC-infection.

### ELISA

The serum level of interferon-gamma (IFN-γ), tumor necrosis factor-alpha (TNF-α), interleukin-1 (IL-1), interleukin-10 (IL-10), interleukin-6 (IL-6), as well as the ileum level of secretory immunoglobulin A (IgA) were determined by a commercially available ELISA kit (Jiangsu Yutong Biological Technology Co., Ltd) according to the manufacturer’s instructions (16).

### Real-Time PCR

mRNA was isolated from frozen liquid nitrogen and ground ileum tissues with TRIzol reagent. The expression of β-actin (house-keeping gene) and intestinal immune-associated genes were determined by RT-PCR according to our previous study (17–19). Primers used were designed according to the sus scrofa sequence (Table 2). The relative expression of mRNA was calculated with ΔΔCt = (Ct_Target_–Ct_β –actin_) treatment–(Ct_Target_–Ct_β –actin_) control (20). Relative expression was normalized and expressed as a ratio to the expression in the CON group.

**TABLE 2:** Primers used in this study^1^.

Gene	Sequence (5′–3′)
β-Actin	F: CTGCGGCATCCACGAAACT R: AGGGCCGTGATCTCCTTCTG
IL-1β	F: GCTAACTACGGTGACAACAA R: TCTTCATCGGCTTCTCCACT
IL-6	F: CAAAGCCACCACCCCTAAC R: TCGTTCTGTGACTGCAGCTT
IL-8	F: AGAACTGAGAAGCAACAACAACAG R: CACAGGAATGAGGCATAGATGTAG
IL-10	F: ATGGGCGACTTGTTGCTGAC R: CACAGGGCAGAAATTGATGACA
IL-18	F: TATGCCTGATTCTGACTGTT R: ATGAAGACTCAAACTGTATCT
TNF-α	F: CCACGTTGTAGCCAATGTCA R: CAGCAAAGTCCAGATAGTCG
NF-κB	F: AGCCATTGACGTGATCCAGG R: CGAAATCGTGGGGCACTTTG
MyD88	F: CCAGCATTGAGGACTGCCG R: ACAGACAGTGATGAACCGCA
TLR2	F: TCACTTGTCTAACTTATCATCCTCTTG R: TCAGCGAAGGTGTCATTATTGC
TLR4	F: GCCATCGCTGCTAACATCATC R: CTCATACTCAAAGATACACCATCGG

*^1^F, forward; R, Reverse.*

### Ileal Mucosa Microbiota Analysis

Sequencing procedures and data analyses were performed by a commercial company (Novogene Co., Ltd., Beijing, China). In brief, total genome DNA from ileal mucosa samples was extracted for amplification using the specific primer with the barcode (V3-V4 regions) (17, 21). Then, the sequencing libraries were generated, assessed, and sequenced on Illumina MiSeq Sequencer (18). The raw tags were paired, filtered, and then analyzed using the operational taxonomic unit (OTU) cluster (14, 22). Principal coordinate analysis (PCoA) to unweighted UniFrac distance metric matrices were applied to beta-diversity. Observed species, Shannon, Simpson, Chao1, ACE, Goods coverage, and PD whole tree were performed to determine alpha-diversity. Beta-diversity and alpha-diversity were used to evaluate the complexity of species diversity. The bacterial relative abundance at the phylum, order, and genus levels was further compared between the four groups; the top 10 most abundant families were defined as dominant genera flora and compared them, respectively. OTUs were then performed for the genome prediction of microbial communities by Phylogenetic Investigation of Communities by Reconstruction of Unobserved States (PICRUSt).

### Ileal Mucosa Untargeted Metabolomic Analyses

Ileal mucosa was separately pestle with liquid nitrogen and the homogenate was resuspended with pre-chilled 80% methanol through the vortex. The samples were incubated on ice for 5 min and subsequently centrifuged at 15,000 *g* at 4°C for 20 min. The supernatants were further transferred to a new Eppendorf tube, centrifuged at 15,000 *g* at 4°C for 20 min, and injected into the LC-MS/MS system analysis (Novogene Co., Ltd., Beijing, China) (23). Data were processed, analyzed, and the metabolite identified according to a previous study (24).

### Western Blot

The detailed process of Western blot was described according to our previous study (18). The primary antibodies included NF-κB (bs-0465R, 1:1000, Bioss), p-NFκB (#3033, 1:1000, CST), p38 (ab170099, 1:1000, abcam), p-p38 (ab47363, 1:500, abcam), JNK (#9252, 1:1000, CST), p-JNK (#4668, 1:1000, CST), and β-actin (66009-1-Ig, 1:5000, Proteintech).

### Statistical Analyses

Data in pigs were evaluated using independent samples *t*-test or factorial ANOVA and *p* < 0.05 was taken to indicate statistical significance (IBM SPSS statistics 20 software). The statistical model contained the effects of infect (LB or ETEC), diet (basal or OKG), and their interaction. Data were expressed as mean ± standard error of the mean (SEM). The Pearson correlation analysis was used to measure the correlation between ileal mucosa microbiota and metabolites by using GraphPad Prism 7.

## Results

### Ornithine α-Ketoglutarate Improved Growth Performance, Feces Score, and Serum and Small Intestinal Immunity in Enterotoxigenic *Escherichia coli*–Infected Pigs

**FIGURE 1:**
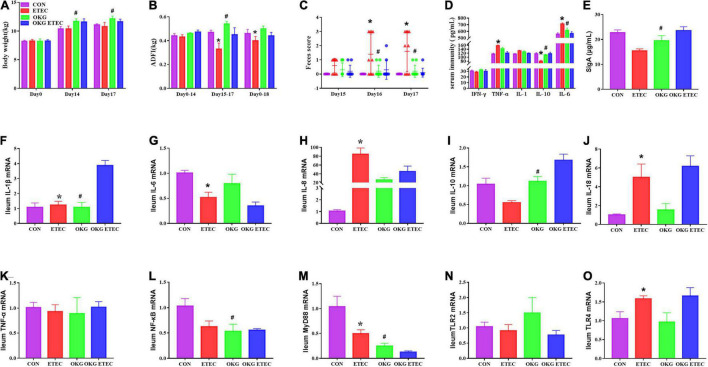
Effects of OKG supplementation on growth performance, feces score, and serum and small intestinal immunity in ETEC- infected pigs. **(A)** Body weight (kg), **(B)** average daily feed intake (kg), **(C)** feces score, **(D)** serum immunity (pg/mL), **(E)** ileum SIgA (μg/ml), **(F–O)** gene expressions of immunity in ileum. Values are presented as mean ± SEM. *Indicates a statistically significant difference for the challenge (ETEC or LB Broth) (*p* < 0.05). ^#^Indicates a statistically significant difference for dietary treatment (basal or OKG diet) (*p* < 0.05).

The growth performance, diarrhea, serum, and small intestinal immunity are shown in Figure 1. The ETEC-challenge significantly decreased average daily feed intake (ADFI) in pigs fed the basal or OKG diet. Compared with the basal diets, OKG increased body weight and ADFI after ETEC-infection (*p* < 0.05). Similarly, ETEC-challenge significantly increased the feces score in pigs fed with the basal or OKG diet. Compared with the CON group, in the OKG group, the feces score was decreased after ETEC-infection (*p* < 0.05). Meanwhile, ETEC-challenge markedly increased the serum TNF-α and IL-6 and decreased the serum IL-10, while OKG treatment markedly increased the serum IL-10 and ileum SIgA and decreased serum IL-6 (*p* < 0.05). We further measured the ileal gene expression of ILs, TNF-α, nuclear factor kappa B (NF-κB), myeloid differentiation primary response gene 88 (MyD88), and transmembrane Toll-like receptors (TLR2 and TLR4) by RT-PCR. ETEC-challenge markedly downregulated the gene expression of IL-1β, IL-6, and MyD88 and improved the mRNA expression of IL-8, IL-18, and TLR4, whereas OKG treatment markedly improved the gene expression of IL-1β and IL-10 and downregulated the mRNA expression of NF-κB and MyD88 (*p* < 0.05).

### Ornithine α-Ketoglutarate Regulated the Ileal Mucosa Microbiota in Enterotoxigenic *Escherichia coli*-Infected Pigs

**FIGURE 2:**
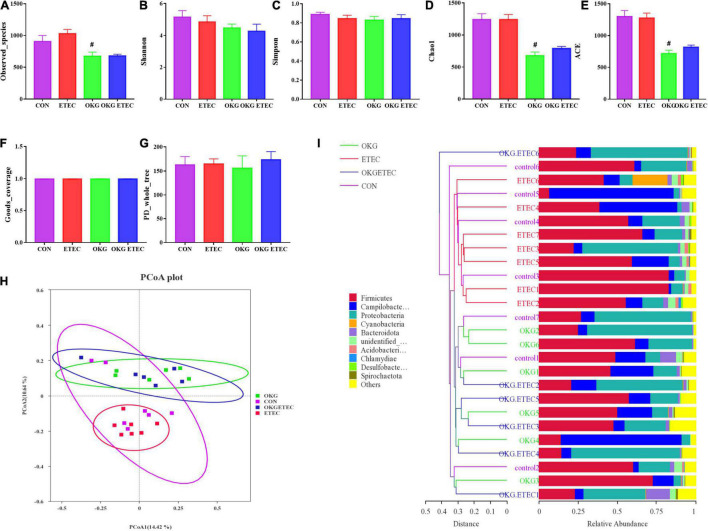
Effects of OKG supplementation on ileal mucosa microbiota diversity in ETEC- infected pigs. **(A–G)** α-diversity analysis, **(H)** PCoA plot analysis from each sample, **(I)** weighted UniFrac distance in each sample. Values are presented as mean ± SEM. *Indicates a statistically significant difference for the challenge (ETEC or LB Broth) (*p* < 0.05). ^#^Indicates a statistically significant difference for dietary treatment (basal or OKG diet) (*p* < 0.05).

The OKG treatment significantly decreased community richness, including observed species, Chao1, and AEC (Figure 2, *p* < 0.05). Dietary supplementation OKG treatment or not presented a clear separation clustering of microbial community composition in beta-diversity (Figure 2).

**FIGURE 3:**
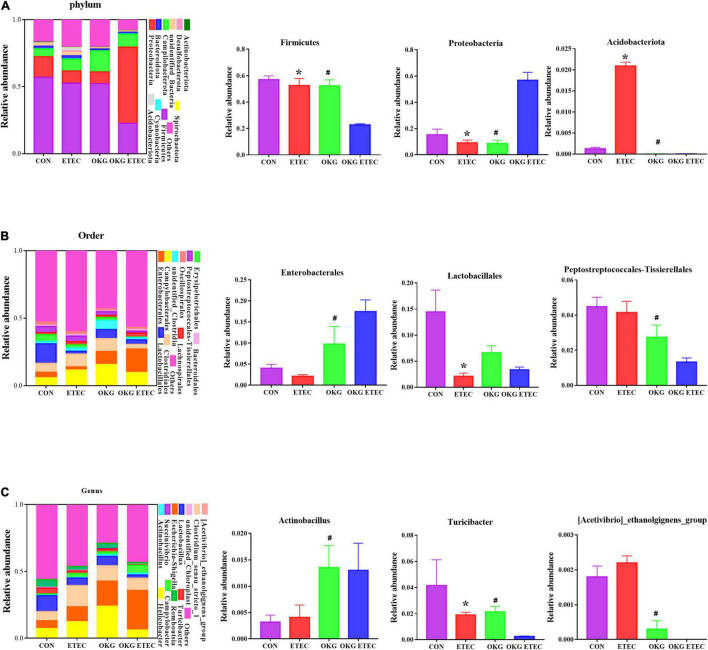
Effects of OKG supplementation on ileal mucosa microbiota compositions at phylum, order, and genus level in ETEC-infected pigs. **(A)** Microbiota compositions at the phylum level, **(B)** microbiota compositions at the order level, and **(C)** microbiota compositions at the genus level. Values are presented as mean ± SEM. *Indicates a statistically significant difference for the challenge (ETEC or LB Broth) (*p* < 0.05). ^#^Indicates a statistically significant difference for dietary treatment (basal or OKG diet) (*p* < 0.05).

The overall microbial composition at the phylum, order, and genus levels is presented in Figure 3. *Firmicutes*, *Campylobacterales*, and *Escherichia-Shigella* were the predominant flora in phylum, order, and genus level, respectively. ETEC-challenge markedly increased *Acidobacteriota*, while OKG treatment markedly reversed these alterations (*p* < 0.05). OKG ETEC groups had a higher proportion of *Proteobacteria*, but a lower proportion of *Firmicutes* (*p* < 0.05). At the order levels, *Lactobacillales* were decreased in relative abundance by ETEC-challenged pigs (*p* < 0.05). OKG treatment markedly increased the proportion of *Enterobacterales* but decreased the proportion of *Peptostreptococcales-Tissierellales*. Similar alterations were observed for *Actinobacillus*, *[Acetivibrio]_cthanolgignens_group*, and *Turicibacter* at the genus level (*p* < 0.05).

**FIGURE 4:**
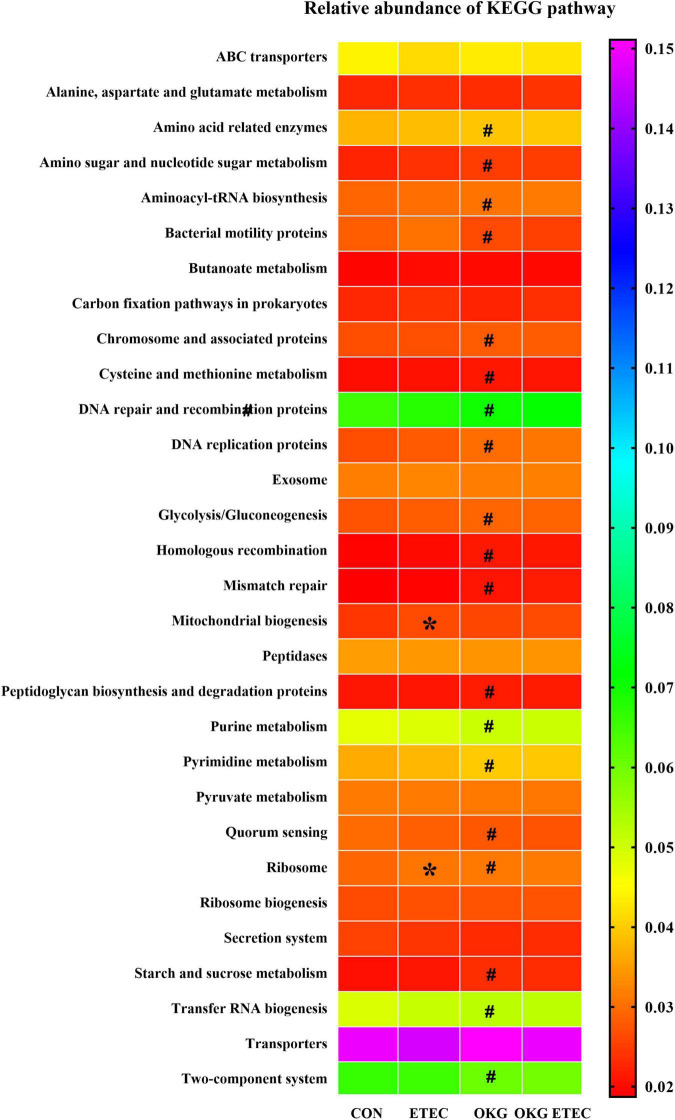
Predictive functional profiling of microbial communities by PICRUSt. KEGG pathway annotations in level 3. Values are presented as mean ± SEM. *Indicates a statistically significant difference for the challenge (ETEC or LB Broth) (*p* < 0.05). ^#^Indicates a statistically significant difference for dietary treatment (basal or OKG diet) (*p* < 0.05).

Phylogenetic Investigation of Communities by Reconstruction of Unobserved States analysis showed that ETEC treatment markedly increased ribosome and mitochondrial biogenesis (*p* < 0.05, Figure 4). OKG treatment significantly increased amino-acid related enzymes, amino sugar and nucleotide sugar metabolism, aminoacyl-tRNA biosynthesis, chromosome and associated proteins, cysteine and methionine metabolism, DNA repair and recombination proteins, DNA replication proteins, glycolysis/gluconeogenesis, homologous recombination, mismatch repair, peptidoglycan biosynthesis, and degradation proteins, purine metabolism, pyrimidine metabolism, ribosome, starch and sucrose metabolism, and transfer RNA biogenesis, but decreased bacterial motility proteins, quorum sensing, and the two-component system (*p* < 0.05).

### Ornithine α-Ketoglutarate Regulated the Ileal Mucosa Metabolites in Enterotoxigenic *Escherichia coli*-Infected Pigs

**FIGURE 5:**
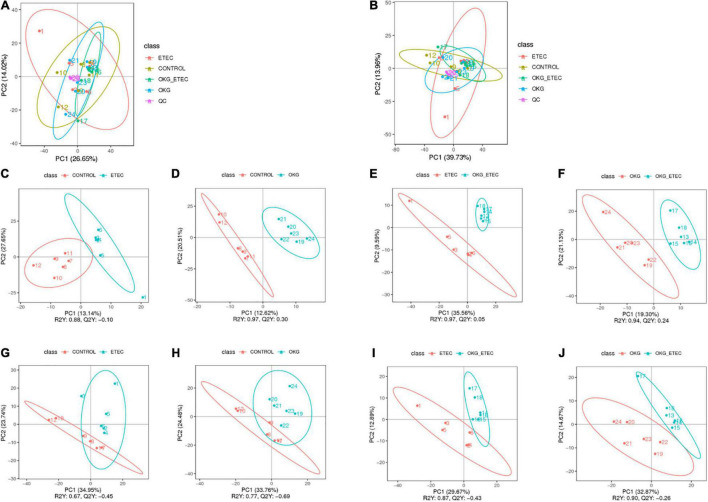
Score plots of principal component analysis (PCA) and Partial least squares discrimination analysis (PLS-DA) models. **(A,B)** PCA based on positive and negative ileal mucosa metabolite profiles. **(C–J)** PLS-DA based on positive and negative ileal mucosa metabolite profiles.

The peaks extracted from all experimental samples and QC samples were analyzed by Principal component analysis (PCA; Figures 5A,B). The QC samples were clustered, which indicated that the data were reliable. Also, the partial least squares discriminant analysis (PLS–DA) score plots presented that ETEC and OKG led to significant metabolite changes (Figures 5C–J).

**FIGURE 6:**
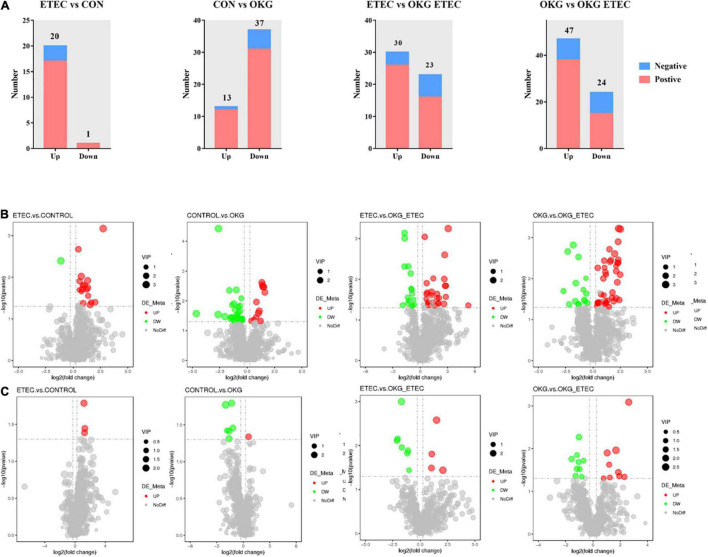
Effects of OKG supplementation on ileal mucosa metabolites in ETEC- infected pigs. **(A)** The number of differentially expressed metabolites with functional annotations (*p* < 0.05 and VIP > 1) in the four groups of piglets. **(B,C)** Volcano plots in differential metabolites, red or green spots indicate a statistically significant up-regulation or down-regulation in the four pairwise comparisons (ETEC vs. CON, CON vs. OKG, ETEC vs. OKG ETEC, and OKG vs. OKG ETEC).

**FIGURE 7:**
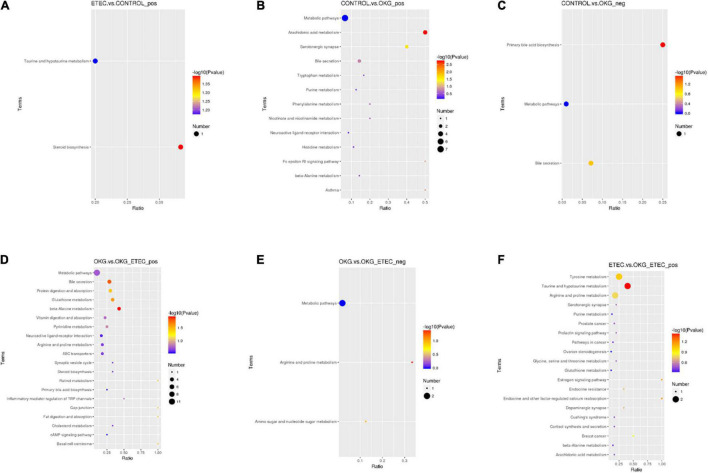
Effects of OKG supplementation on KEGG pathway enrichment in ETEC- infected pigs. **(A)** ETEC vs. CON on positive mode. **(B–C)** CON vs. OKG on positive and negative mode. **(D–E)** OKG vs. OKG ETEC on positive and negative mode. **(F)** ETEC vs. OKG ETEC on positive mode.

Metabolome analyzed a total of 156 positive ions and 39 negative ions with functional annotations. Among them, 21 (20 up and 1 down), 50 (13 up and 37 down), 53 (30 up and 234 down), and 71 (47 up and 24 down) metabolites with functional annotations were significantly different (*p* < 0.05 and VIP > 1) in the four pairwise comparisons (ETEC vs. CON, CON vs. OKG, ETEC vs. OKG ETEC, and OKG vs. OKG ETEC; Figure 6A). Their *m/z*, VIP values, and *p*-values are listed in Supplementary Table 1. In Figures 6B,C, the volcano plots showed the differentially expressed metabolites in the comparisons. Next, the Kyoto Encyclopedia of Genes and Genomes (KEGG) was used to analyze the pathways of the metabolites that differed between the two groups (Figure 7). Compared with the control group, the main metabolic pathways in the OKG group that were enriched are arachidonic acid metabolism, serotonergic synapse, and primary bile acid biosynthesis, and the main metabolic pathways in the ETEC group that were enriched are steroid biosynthesis (*P* < 0.05). Compared with the OKG ETEC group, the main metabolic pathways in the OKG group that were enriched are beta-alanine metabolism, bile secretion, glutathione metabolism, protein digestion and absorption, and arginine and proline metabolism, and the main metabolic pathways in the ETEC group that were enriched are taurine and hypotaurine metabolism (*p* < 0.05).

Based on the comparison of the data with KEGG, 14 identified endogenous metabolites were characterized. These metabolites were ergocalciferol, leukotriene C4, 16(R)-HETE, thromboxane B2, chenodeoxycholic acid, sulfoacetic acid, hypotaurine, spermidine, tyramine, spermine, cholesterol, L-histidine, cadaverine, serotonin, and D-proline. Among these metabolites, ergocalciferol was increased significantly in the ETEC pigs, and leukotriene C4, 16(R)-HETE, thromboxane B2, chenodeoxycholic acid were increased clearly in the OKG pigs compared to the controls (*p* < 0.05). Compared with the OKG ETEC group, sulfoacetic acid and hypotaurine were clearly increased in the ETEC pigs, and spermidine, tyramine, cholesterol, L-histidine, cadaverine, serotonin were significantly increased, while spermine and D-proline were markedly decreased in the OKG group (*p* < 0.05).

### Correlation Analyses Between Ileal Mucosa Microbiota and Metabolites

**FIGURE 8:**
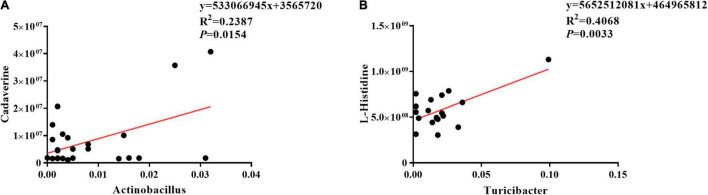
Correlation analyses between ileal mucosa microbiota and metabolites. **(A)** Cadaverine vs. *Actinobacillus*, **(B)** L-histidine vs. *Turicibacter*.

The results of Pearson correlation (*r*) between metabolites and microbiota are shown in Figure 8. We found different levels of correlation in two groups of correlation analyses (*p* < 0.05): cadaverine vs. *Actinobacillus* (*R*^2^ = 0.2387; *p* = 0.0154; Figure 8A) and L-histidine vs. *Turicibacter* (*R*^2^ = 0.4068; *p* = 0.0033; Figure 8B).

### Ornithine α-Ketoglutarate Supplementation on Inflammation-Related Signaling in Enterotoxigenic *Escherichia coli*-Infected Piglets

**FIGURE 9:**
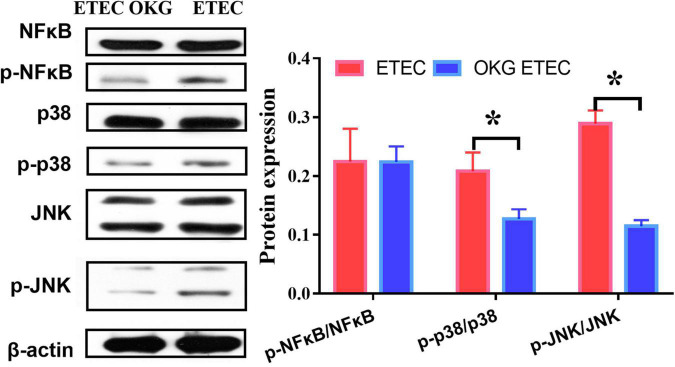
Effects of OKG supplementation on NF-κB/JNK inflammatory pathways in ETEC infected pigs. Values are presented as mean ± SEM. *Indicates a statistically significant difference for the challenge (ETEC or LB Broth) (*p* < 0.05).

Ornithine α-ketoglutarate treatment failed to affect NF-κB expression in ETEC-challenged pigs. We further determined several downstream signals of NF-κB (i.e., JNK and p38). The results indicated that OKG treatment suppressed the phosphorylation of JNK and p38 (*p* < 0.05, Figure 9).

## Discussion

Enterotoxigenic *Escherichia coli* is considered to be a potential pathogen threatening human and animal diarrhea, resulting in huge economic losses (25). ETEC triggers inflammation and intestinal dysfunction, ultimately leading to diarrhea (26). To induce pig diarrhea at least 10^9^ to 10^10^ ETEC is required that is generally obtained 1–5 days after ETEC infection (27). The nutritional supplement can reduce ETEC infection (20). In detail, arginine or glutamine supplementation suppresses ETEC colonization in the ETEC-challenged mouse (28). Our group and others have previously demonstrated that OKG changes or reverses growth performance, intestinal microbes, and immunity in animal models (14, 29). OKG is a nutrient compound obtained from ornithine and α-ketoglutarate (30), which is the precursor of glutamine, arginine, proline, and polyamines (12). However, there is no direct evidence that OKG mediated gut microbiota and immunity in ETEC-infected pig models based on previous reports. Thus, in the current study, we focused on OKG to prevent ETEC-infection diarrhea *via* regulation of immunity, alterations of ileal mucosa microbiota and metabolites. Notably, growth performance was inhibited in ETEC infection, and OKG supplementation improved growth performance, which is similar to the effect of OKG on pigs induced with D-galactose chronic oxidative stress and OKG on tumor-bearing rats (14, 29). In ETEC K88-infection pigs, the day immediately after inoculation, a clear peak of diarrhea could also be observed in infected animals, and Chito-oligosaccharide reduced the incidence of diarrhea (1). Similarly, our study found that OKG supplementation markedly reduced the feces score after ETEC infection, which is in accordance with previous reports.

Pro-inflammatory cytokines such as IFN-γ, TNF-α, IL-1, and IL-6 are measured as biomarkers for gastrointestinal disorders, including ETEC-infection diarrhea (31). An NF-κB signaling pathway is associated with inflammation (26). Intermittent fasting can inhibit the NF-κB/JNK inflammatory pathways and thus reduce inflammation and alleviate type-2 diabetes symptoms (32). Our results also demonstrate that ETEC-infection increases the serum TNF-α, and IL-6, while OKG can significantly decrease the serum IL-6 and suppress the phosphorylation of downstream signals of NF-κB/JNK. Thus, the anti-inflammatory effect of OKG may be associated with suppressing the NF-κB pathway. IL-10 can inhibit the production of other inflammatory factors by blocking the activation of NF-κB and is an important regulator of the immune response (33, 34). SIgA is the main part of the intestinal immunological barrier and the most abundant immunoglobulin in the body, which also can clear pathogenic microorganisms (6, 35, 36). Nucleotides supplementation promotes the development of small intestinal villus and secretory IgA in neonatal piglets, prevents diarrhea, and increases the weaning weight of piglets (19). Meantime, we found similar results that OKG supplementation can increase the ileum SIgA and serum IL-10 secretion in piglets.

Chitosan supplementation enhances the gene expression of IL-1β and IL-6 in the intestine compared with ETEC, alleviates intestinal inflammation, and enhances the cell-mediated immune response (1). ETEC-infection can suppress the gene expression of pro-inflammatory cytokines, including IL-1β, IL-6, IL-8, TNF-α, and IL-17A, indicating ETEC infection inhibits the inflammatory reaction (37). In the neonatal piglets, nucleotides upregulate the gene expression of IL-17, IL-8, IL-6, IL-1β, IL-10, and TNF-α in the intestine, prevent diarrhea, and increase the weaning weight of piglets (19). Meanwhile, pathogen-associated molecular patterns adaptors include MyD88 (38). Asp supplementation suppresses TLRs and MyD88 expression in LPS-induced weaned pigs (39). Similarly, our experimental results indicate that ETEC-infection downregulates the mRNA expression of IL-1β, IL-6, and MyD88 and improves the mRNA expression of IL-8, IL-18, and TLR4, while OKG supplementation increases the gene expression of IL-1β and IL-10 and reduces the NF-κB and MyD88 in the ileum. However, OKG supplementation fails to affect NF-κB expression, and it only suppresses the phosphorylation of downstream signals of NF-κB/JNK. mRNA–protein relation is generally linear; however, mRNA to protein *in vivo* involves transcription, translation, and the turnover of proteins, and thus the determinants of protein abundance (40, 41). These observations suggested that OKG may alleviate intestinal inflammation and prevent diarrhea when ETEC infection represses the intestinal inflammatory response

The gastrointestinal microbes act as the first line against harmful endogenous and exogenous substances entering the body (42–45). The homeostasis between the host and gastrointestinal microbiota is disturbed so that it results in the incidence of diarrhea (26). OKG administration can prevent bacterial dissemination so that endotoxemia is reduced (46). In ETEC infection-induced diarrhea piglet model, the diversity, structure, and function of the gut microbial community are decreased and ETEC could induce diarrhea *via* intestinal microbiota in piglets (47). However, OKG groups change the diversity, structure, and function of the gut microbial community, including Observed species, Chao1 and ACE, decreased and difference in beta-diversity presented difference. The reason may be that the substantially higher relative abundance of *Enterobacterales*, which is consistent with OKG, suppressed alpha diversity in our previous study (14). At the genus level, OKG increased *Actinobacillus*, but decreased *Turicibacter* and *[Acetivibrio]_ethanolgignens_group*. Intestinal fora *[Acetivibrio]_ethanolgignens_group* and *Turicibacter* are involved in immune function (47, 48). *[Acetivibrio]_ethanolgignens_group* can induce metabolic disorder and liver inflammation (48). *Turicibacter* has adverse effects on intestinal health and lipid metabolism of the host (49, 50). Additionally, increased *Actinobacillus* can impair barrier function (51). However, OKG supplementation failed to reduce the *Actinobacillus* abundance. The reason may be that OKG decreased other pro-inflammatory bacteria, including *Turicibacter* and *[Acetivibrio]_ethanolgignens_group*, thus improving growth performance and regulating immunity. Predictive functional profiling of microbial communities further confirms that altered microbiota mainly involves genes related to the ribosome and mitochondrial biogenesis, amino acid-related enzymes, amino sugar and nucleotide sugar metabolism, aminoacyl-tRNA biosynthesis, chromosome and associated proteins, cysteine and methionine metabolism, DNA repair and recombination proteins, DNA replication proteins, glycolysis/gluconeogenesis, homologous recombination, mismatch repair, peptidoglycan biosynthesis and degradation proteins, purine metabolism, pyrimidine metabolism, ribosome, starch and sucrose metabolism, and transfer RNA biogenesis, but decreased bacterial motility proteins, quorum sensing, and two-component system. These suggested that OKG prevents ETEC-infection diarrhea *via* alterations of the ileal mucosa microbiota.

The present study also determined ileal mucosal microbe-derived metabolites consistent with changes in the ileal mucosa microbiome. OKG increased leukotriene C4, 16(R)-HETE, thromboxane B2, and chenodeoxycholic acid. Leukotriene C4, 16(R)-HETE, and thromboxane B2, a product of arachidonic acid, can improve anti-inflammation, enhance intestinal health, and cure disease (52–55). For instance, 16(R)-HETE can suppress the activation of polymorphonuclear leukocytes and reduce intracranial pressure (53). Chenodeoxycholic acid, which is involved in primary bile acid biosynthesis, inhibits pro-inflammatory cytokines (56). These suggested that OKG regulates immune status and alleviates the suppression in piglets infected by ETEC *via* stimulating the ileal mucosa microbe and metabolites. Our data indicate that the cadaverine level was positively correlated with *Actinobacillus* and L-histidine level was positively correlated with *Turicibacter* abundance.

## Conclusion

In conclusion, dietary supplementation with 1% OKG regulates serum levels of TNF-α, IL-6, and IL-10, alters the genes expression related to ileal immunity, and alleviates the growth-suppression of piglets infected by ETEC, which may be associated with positive alterations in the composition of gut microbiota and modulation in the gut metabolites, especially for *Actinobacillus*, *[Acetivibrio]_ethanolgignens_group*, and *Turicibacter*. Further studies with the use of OKG in healthy animals and clinical application are anticipated.

## Data Availability Statement

The datasets presented in this study can be found in online repositories. The names of the repository/repositories and accession number(s) can be found below: NCBI, PRJNA808781.

## Ethics Statement

The animal study was reviewed and approved by Committee on Animal Care of the Institute of Subtropical Agriculture, Chinese Academy of Sciences (Changsha, CAS20190409). Written informed consent was obtained from the owners for the participation of their animals in this study.

## Author Contributions

YL and KY conceived the study. YL wrote the original draft of the manuscript. All authors have contributed to the development of the methodology, design of the study, reviewed, and edited the manuscript.

## Conflict of Interest

The authors declare that the research was conducted in the absence of any commercial or financial relationships that could be construed as a potential conflict of interest.

## Publisher’s Note

All claims expressed in this article are solely those of the authors and do not necessarily represent those of their affiliated organizations, or those of the publisher, the editors and the reviewers. Any product that may be evaluated in this article, or claim that may be made by its manufacturer, is not guaranteed or endorsed by the publisher.

## Supplementary Material

Supplementary Data Sheet 1 Effects of OKG supplementation on ileal mucosa differential metabolites in ETEC- infected pigs.
